# Development and validation of a prognostic nomogram for overall survival in patients with primary cutaneous T-cell lymphoma: A SEER-based study

**DOI:** 10.1097/MD.0000000000048761

**Published:** 2026-05-08

**Authors:** Rongli Xie, Mei Xie, Xiaojun Xu, Tiantian Sun

**Affiliations:** a Department of Hematology, The Seventh Affiliated Hospital, Sun Yat-Sen University, Shenzhen, Guangdong, China.

**Keywords:** cutaneous T-cell lymphoma, incidence, nomogram, prognosis, SEE

## Abstract

The prognosis of cutaneous T-cell lymphoma (CTCL) remains heterogeneous, and clinically applicable prognostic tools are needed. Using population-based Surveillance, Epidemiology, and End Results (SEER) data (2000–2019), we analyzed incidence trends and developed a prognostic nomogram for overall survival. Incidence rates were assessed by joinpoint regression. Patients diagnosed from 2004 to 2010 formed the training cohort (n = 4316), and those from 2011 to 2015 constituted the temporal validation cohort (n = 2875). Multivariable Cox analysis identified independent prognostic factors, which were incorporated into a nomogram predicting 1-, 3- and 5-year overall survival. Among 7191 patients, CTCL incidence increased from 2000 to 2016 [annual percent change (APC) 1.05%] and then declined through 2019 (APC −8.44%). The nomogram integrates 11 variables: age, sex, race, marital status, histological subtype, median household income, TNM stage, prior malignances, surgery, radiation, and chemotherapy. It demonstrated strong discrimination, with time-dependent area under the curve values >0.815 for all timepoints in both cohorts, and good calibration. Based on nomogram-derived scores, patients were stratified into 3 distinct risk groups with significantly different survival (*P* < .0001). This SEER-based nomogram provides a practical, multivariable tool for risk stratification in CTCL, using routinely available clinical and demographic data. External validation is warranted to confirm its generalisability.

## 1. Introduction

Cutaneous T-cell lymphoma (CTCL) comprises a heterogeneous group of non-Hodgkins lymphomas with diverse clinical behavior and outcomes.^[1]^ While the TNM staging system provides anatomical tumor assessment,^[2,3]^ its prognostic accuracy remains suboptimal for clinical decision-making due to limited incorporation of key demographic and socioeconomic variables.^[2,4]^ Existing prognostic indices such as the Cutaneous Lymphoma International Prognostic Index offer improved risk stratification for mycosis fungoides and Sézary syndrome.^[5,6]^ However, their application is restricted to specific subtypes and they lack comprehensive integration of routinely available clinicodemographic parameters.^[7,8]^

To address these limitations, this study aimed to develop and validate a prognostic nomogram for overall survival (OS) in patients with CTCL using population-based Surveillance, Epidemiology, and End Results (SEER) data. The nomogram integrates multiple prognostic domains – including demographic characteristics, disease parameters, socioeconomic factors and treatment modalities – across the full spectrum of CTCL subtypes, in order to provide individualized survival estimates at diagnosis. Additionally, we analyzed recent incidence trends to provide relevant background for the prognostic model.

## 2. Materials and methods

### 2.1. Data source and patient selection

We collected the incidence and survival data for CTCL from the SEER 18 Registries Database (2000–2019) using SEER*Stat software (version 8.3.6). Cases were identified using ICD-O-3 morphology codes 9700 to 9702, 9705, 9708, 9709, 9714, 9718, 9719, 9726, 9827 and topography codes C44.0 to C44.9.

Patients with microscopically confirmed CTCL diagnosed between 2000 and 2015 and with ≥4 years of follow-up were included. We excluded cases diagnosed by autopsy or death certificate only, and those with missing data on race, marital status, TNM stage, or treatment. A total of 8351 patients (53.7% of initially identified cases) were excluded due to incomplete data. The missing data patterns were as follows: TNM stage was missing in 41.2% of initially identified cases, histologic subtype in 28.7%, and treatment variables (surgery, radiation, chemotherapy) in 19.3%. Among excluded patients, approximately 30% had both stage and histology missing, while others had missing data on one or more variables. Comparative analysis showed that excluded and included cohorts had similar distributions of age (median 61 vs 62 years), sex (56.2% vs 57.8% male), and marital status (58.4% vs 59.1% married). Due to the nature of missingness – key prognostic variables (stage and histology) were precisely the fields missing for the excluded patients – a direct comparison of these factors between the 2 groups was not feasible. Missing data were handled using complete-case analysis.

This substantial exclusion introduces a potential selection bias that we cannot fully quantify. While the available demographic data were comparable between cohorts, the missingness of key prognostic variables limits our ability to assess the representativeness of the included cohort. This limitation is acknowledged as a primary constraint in interpreting our findings (see Section 4).

### 2.2. Statistical analysis

After selection, 7191 patients were included. Join-point regression (version 4.5.0.1) analyzed incidence trends. Survival analysis used Kaplan–Meier method with log-rank tests. Cox proportional hazards model estimated hazard ratios (HRs) with 95% confidence intervals (CIs). The proportional hazards assumption was tested using scaled Schoenfeld residuals; all predictors satisfied the assumption (global test *P* > .05).

For model development, patients were divided into training (2004–2010, n = 4316) and validation (2011–2015, n = 2875) cohorts. This temporal split evaluates model performance on more recent patients, assessing its applicability to future cases and enhancing external validity.

Baseline characteristics were compared using Chi-squared or Fisher exact tests for categorical variables and Mann–Whitney *U* test for continuous variables. Restricted cubic spline analysis with 4 knots examined nonlinear age-OS relationships. Statistical analyses used R (version 4.0.5) with specific packages: Cox regression (“survival”), time-dependent AUC (“timeROC”), and calibration plots (“rms”).

Risk stratification used X-tile software (version 3.6.1), which determines optimal cutpoints by maximizing the log-rank statistic for survival differences between groups, ensuring statistically robust risk categorization.

To assess potential overfitting, we performed internal validation using 500 bootstrap resamples to estimate optimism-corrected performance.

### 2.3. Bias considerations

As a registry-based study, potential biases include selection bias (SEER covers ~28% of US population) and confounding bias (missing data on comorbidities, detailed treatments, and laboratory values). We mitigated these by adjusting for available clinicopathological/socioeconomic variables and using temporal validation. The comparable characteristics between excluded and included patients suggest limited selection bias.

### 2.4. Sensitivity analysis considerations

We recognize that sensitivity analyses (e.g., models excluding treatment variables, multiple imputation) would further strengthen the methodological rigor. However, such analyses were not feasible due to 2 key constraints: the excluded patients lack the essential variables (TNM stage, histologic subtype) required for meaningful sensitivity analyses; and our access to the SEER database for additional data extraction is no longer available. Any imputation or assumption-based approach would introduce substantial uncertainty and could itself be methodologically questionable. Instead, we have addressed these concerns through enhanced transparency in reporting missing data patterns, explicit Acknowledgments of limitations, and careful framing of variable interpretation throughout the manuscript.

## 3. Results

### 3.1. Incidence trend of CTCL

**Figure 1.**
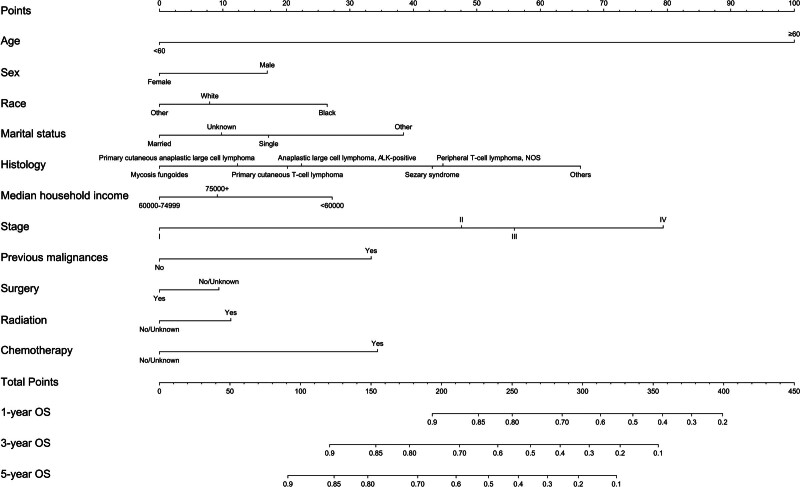
Incidence trend of CTCL from 2000 to 2019 adjusted to the 2000 US standard population. CTCL = cutaneous T-cell lymphoma.

The overall CTCL incidence rate increased from 2000 to 2016 (annual percent change [APC], 1.05%) and subsequently decreased from 2017 to 2019 (APC, −8.44%) (Fig. 1). This pattern may reflect evolving diagnostic criteria and improved disease recognition in recent years.

### 3.2. Patient characteristics

The study included 7191 CTCL patients with balanced baseline characteristics between training (n = 4316) and validation (n = 2875) cohorts (Tables 1–3). The predominance of early-stage disease (stage I: 42.3%) supports the characteristically indolent nature of most CTCL subtypes at diagnosis.

**Table 1:** Patient demographic characteristics in the training cohort and external validation cohort.

	ALL	Training	Validation	*P*-overall
	N = 7191	N = 4316	N = 2875	
Sex				
Female	3005 (41.8%)	1813 (42.0%)	1192 (41.5%)	.663
Male	4186 (58.2%)	2503 (58.0%)	1683 (58.5%)	
Age	61.0 [48.0; 72.0]	61.0 [48.0; 72.0]	61.0 [47.0; 71.0]	.569
Race				
White	5665 (78.8%)	3397 (78.7%)	2268 (78.9%)	.933
Black	1013 (14.1%)	613 (14.2%)	400 (13.9%)	
Other	513 (7.13%)	306 (7.09%)	207 (7.20%)	
Marital status				
Married	3663 (50.9%)	2203 (51.0%)	1460 (50.8%)	.782
Single	1239 (17.2%)	755 (17.5%)	484 (16.8%)	
Other	1000 (13.9%)	598 (13.9%)	402 (14.0%)	
Unknown	1289 (17.9%)	760 (17.6%)	529 (18.4%)	
Median household income (renminbi)				
<60,000	1764 (24.5%)	1069 (24.8%)	695 (24.2%)	.742
–74,999	2882 (40.1%)	1715 (39.7%)	1167 (40.6%)	
75,000+	2545 (35.4%)	1532 (35.5%)	1013 (35.2%)	
Residence				
Rural	594 (8.26%)	350 (8.11%)	244 (8.49%)	.599
Urban	6597 (91.7%)	3966 (91.9%)	2631 (91.5%)	
No/Unknown	5363 (74.6%)	3225 (74.7%)	2138 (74.4%)	

**Table 2:** Patient disease and treatment characteristics in the training cohort and external validation cohort.

	ALL	Training	Validation	*P*-overall
	N = 7191	N = 4316	N = 2875	
Histology				
Mycosis fungoides	3787 (52.7%)	2286 (53.0%)	1501 (52.2%)	.462
Primary cutaneous T-cell lymphoma	1740 (24.2%)	1046 (24.2%)	694 (24.1%)	
Primary cutaneous anaplastic large cell lymphoma	800 (11.1%)	490 (11.4%)	310 (10.8%)	
Peripheral T-cell lymphoma, not otherwise specified	461 (6.41%)	268 (6.21%)	193 (6.71%)	
Anaplastic large cell lymphoma, anaplastic lymphoma kinase-positive	151 (2.10%)	90 (2.09%)	61 (2.12%)	
Sezary syndrome	138 (1.92%)	77 (1.78%)	61 (2.12%)	
Others	114 (1.59%)	59 (1.37%)	55 (1.91%)	
Stage				
I	5482 (76.2%)	3304 (76.6%)	2178 (75.8%)	.392
II	533 (7.41%)	316 (7.32%)	217 (7.55%)	
III	365 (5.08%)	204 (4.73%)	161 (5.60%)	
IV	811 (11.3%)	492 (11.4%)	319 (11.1%)	
Previous malignances				
No	5948 (82.7%)	3564 (82.6%)	2384 (82.9%)	.728
Yes	1243 (17.3%)	752 (17.4%)	491 (17.1%)	
Surgery				
No/Unknown	5155 (71.7%)	3108 (72.0%)	2047 (71.2%)	.471
Yes	2036 (28.3%)	1208 (28.0%)	828 (28.8%)	
Radiation				
No/Unknown	5692 (79.2%)	3435 (79.6%)	2257 (78.5%)	.281
Yes	1499 (20.8%)	881 (20.4%)	618 (21.5%)	
Chemotherapy				
No/Unknown	5363 (74.6%)	3225 (74.7%)	2138 (74.4%)	.755
Yes	1828 (25.4%)	1091 (25.3%)	737 (25.6%)	

**Table 3:** Univariate and multivariate analysis of overall survival in the training cohort.

Variable	All	HR (univariable)	HR (multivariable)
Age_group2			
<60	2061 (47.8%)		
≥60	2255 (52.2%)	4.18 (3.69–4.73, *P* < .001)	3.77 (3.30–4.31, *P* < .001)
Sex			
Female	1813 (42.0%)		
Male	2503 (58.0%)	1.20 (1.08–1.34, *P* < .001)	1.25 (1.12–1.40, *P* < .001)
Race			
White	3397 (78.7%)		
Black	613 (14.2%)	1.12 (0.97–1.29, *P* = .12)	1.27 (1.09–1.48, *P* = .002)
Other	306 (7.1%)	0.64 (0.50–0.82, *P* < .001)	0.90 (0.70–1.16, *P* = .41)
Marital status			
Married	2203 (51.0%)		
Single	755 (17.5%)	0.88 (0.75–1.03, *P* = .12)	1.26 (1.07–1.48, *P* = .006)
Other	598 (13.9%)	2.17 (1.90–2.47, *P* < .001)	1.66 (1.44–1.91, *P* < .001)
Unknown	760 (17.6%)	0.88 (0.76–1.03, *P* = .11)	1.14 (0.97–1.33, *P* = .11)
Histology			
Mycosis fungoides	2286 (53.0%)		
Primary cutaneous T-cell lymphoma	1046 (24.2%)	1.57 (1.39–1.78, *P* < .001)	1.31 (1.15–1.49, *P* < .001)
Primary cutaneous anaplastic large cell lymphoma	490 (11.4%)	1.34 (1.14–1.59, *P* < .001)	1.18 (0.99–1.40, *P* = .07)
Peripheral T-cell lymphoma, not otherwise specified	268 (6.2%)	2.48 (2.06–2.98, *P* < .001)	1.81 (1.49–2.19, *P* < .001)
Anaplastic large cell lymphoma, anaplastic lymphoma kinase-positive	90 (2.1%)	1.27 (0.88–1.81, *P* = .20)	1.34 (0.92–1.93, *P* = .12)
Sezary syndrome	77 (1.8%)	4.89 (3.71–6.43, *P* < .001)	1.77 (1.32–2.38, *P* < .001)
Others	59 (1.4%)	3.45 (2.44–4.87, *P* < .001)	2.41 (1.69–3.45, *P* < .001)
Median household income			
<60,000	1069 (24.8%)		
–74,999	1715 (39.7%)	0.62 (0.55–0.71, *P* < .001)	0.68 (0.60–0.78, *P* < .001)
75,000+	1532 (35.5%)	0.65 (0.57–0.73, *P* < .001)	0.77 (0.67–0.89, *P* < .001)
Residence			
Rural	350 (8.1%)		
Urban	3966 (91.9%)	0.67 (0.57–0.79, *P* < .001)	1.08 (0.90–1.30, *P* = .41)
Stage			
I	3304 (76.6%)		
II	316 (7.3%)	2.18 (1.83–2.59, *P* < .001)	1.88 (1.57–2.25, *P* < .001)
III	204 (4.7%)	3.26 (2.70–3.93, *P* < .001)	2.10 (1.71–2.57, *P* < .001)
IV	492 (11.4%)	3.92 (3.45–4.47, *P* < .001)	2.87 (2.48–3.31, *P* < .001)
Previous malignances			
No	3564 (82.6%)		
Yes	752 (17.4%)	2.29 (2.04–2.57, *P* < .001)	1.56 (1.38–1.75, *P* < .001)
Surgery			
No/Unknown	3108 (72.0%)		
Yes	1208 (28.0%)	0.77 (0.68–0.86, *P* < .001)	0.88 (0.78–1.00, *P* = .05)
Radiation			
No/Unknown	3435 (79.6%)		
Yes	881 (20.4%)	1.37 (1.22–1.54, *P* < .001)	1.16 (1.02–1.32, *P* = .02)
Chemotherapy			
No/Unknown	3225 (74.7%)		
Yes	1091 (25.3%)	2.26 (2.03–2.51, *P* < .001)	1.58 (1.40–1.77, *P* < .001)

n = 4316, events = 1452, Likelihood ratio test = 1373.41 on 23 df (*P* < .001).

Important note regarding clinical management variables: Treatment variables (surgery, radiation, chemotherapy) are included in the model to enhance its clinical predictive utility. It is important to note that their association with survival (e.g., the elevated hazard ratio for chemotherapy in the multivariable analysis) primarily reflects the more aggressive or advanced disease state for which these interventions are typically indicated (i.e., confounding by indication), rather than an independent causal effect of the treatment itself. In this model, these variables should be interpreted as composite markers of disease severity and initial clinical management pathways.

HR = hazard ratio.

### 3.3. Survival outcomes

**Figure 2.**
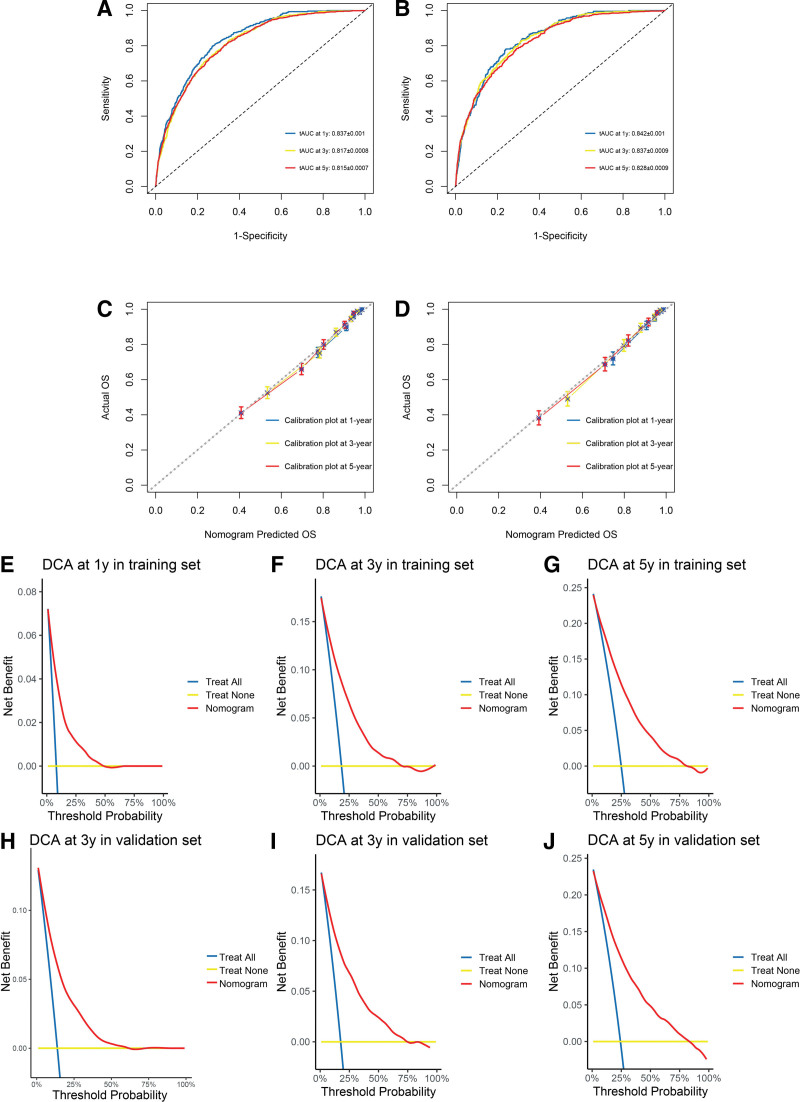
Overall survival analysis of CTCL according to years of diagnosis and cohort. CTCL = cutaneous T-cell lymphoma.

OS did not differ significantly by diagnosis year or between training and validation cohorts (Fig. 2), supporting the appropriateness of our temporal validation approach.

### 3.4. Prognostic factors and clinical implications

Multivariate Cox analyses identified eleven independent prognostic factors (Table 3). The observed associations of marital status and household income with survival may be mediated through complex sociobehavioural and healthcare-access pathways, such as differences in social support systems or ability to engage with long-term care. While these non-biological factors are important for holistic patient management, their precise mechanisms in the context of CTCL require further prospective investigation.

**Figure 3.**
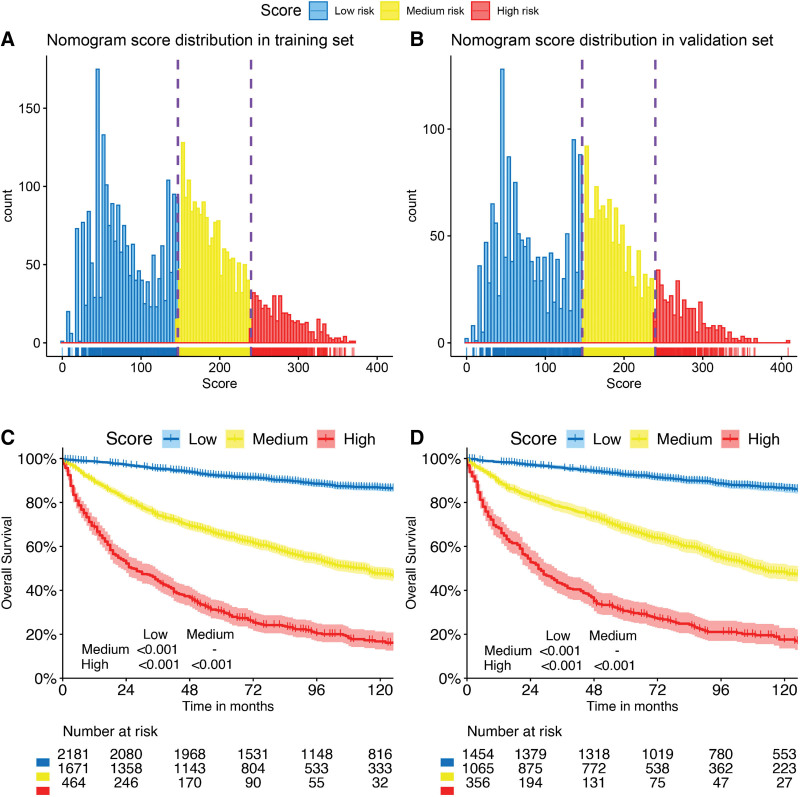
Restricted cubic spline is used to flexibly model and visualize the association between age and HR for OS. HR = hazard ratio, OS = overall survival.

Age demonstrated a nonlinear relationship with mortality, with significantly worsened prognosis beyond 60 years (Fig. 3), with a markedly worse prognosis beyond 60 years (Fig. 3).

Interpretation of the treatment variables (surgery, radiation, chemotherapy) warrants specific caution. Their inclusion in the model captures initial management patterns, but the associations should not be construed as causal. For instance, the significant association between chemotherapy and inferior survival (HR 1.58) is consistent with its application in patients with higher baseline disease burden and more aggressive features. Within this prognostic framework, these variables are best understood as proxy indicators for underlying disease severity and the clinical pathways it necessitates. They should be viewed primarily as indicators of advanced disease severity rather than independent prognostic factors.

### 3.5. Nomogram performance and clinical utility

**Figure 4.**
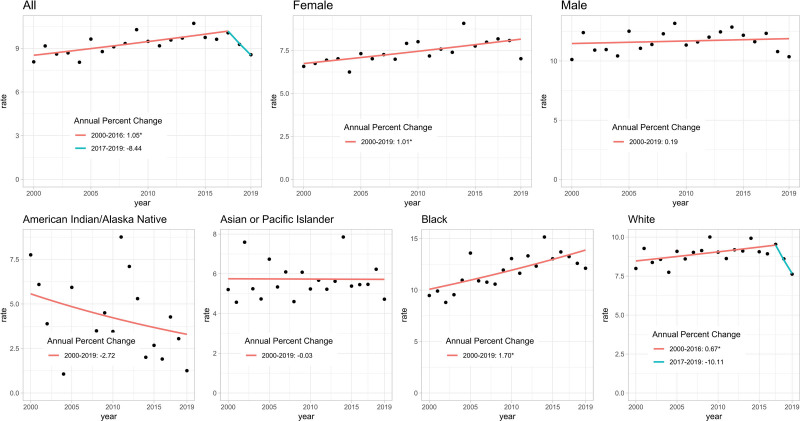
Nomogram to predict the probabilities of 1-, 3-, and 5-yr overall survival for CTCL. CTCL = cutaneous T-cell lymphoma.

**Figure 5.**
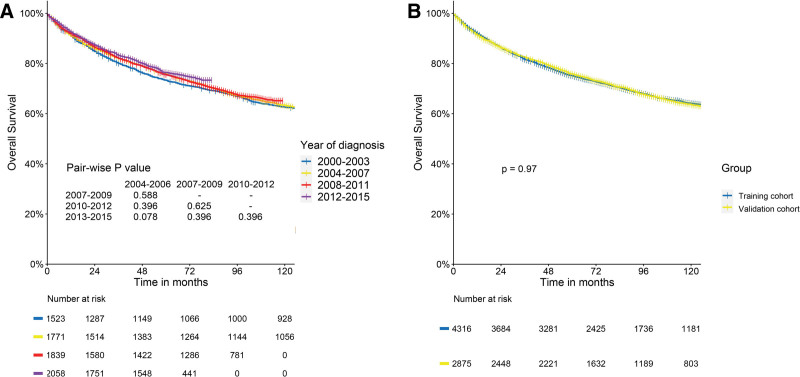
Evaluation of the discrimination (A, B), calibration (C, D), and clinical usefulness (E–J) of the nomogram. (A) The 1-, 3-, and 5-yr ROC curves and AUC values in the training cohort (n = 4316) with AUC values of 0.837 (95% CI: 0.820–0.854), 0.817 (95% CI: 0.803–0.831), and 0.815 (95% CI: 0.800–0.830), respectively. (B) The 1-, 3-, and 5-yr ROC curves and AUC values in the validation cohort (n = 2875) with AUC values of 0.842 (95% CI: 0.822–0.862), 0.837 (95% CI: 0.820–0.854), and 0.828 (95% CI: 0.810–0.846), respectively. The calibration plots at 1, 3, and 5 yr in the (C) training and (D) validation cohorts. The decision curve analyses at 1 (E, H), 3 (F, I), and 5 (G, J) years in the training and validation cohorts. AUC = area under the time-dependent receiver operating characteristic curve, CI = confidence interval, ROC = receiver operating characteristic.

The prognostic nomogram integrated all identified risk factors (Fig. 4). The model demonstrated strong predictive accuracy with AUC values of 0.837 (95% CI: 0.820–0.854), 0.817 (95% CI: 0.803–0.831), and 0.815 (95% CI: 0.800–0.830) for 1-, 3-, and 5-year OS in the training cohort, and 0.842 (95% CI: 0.822–0.862), 0.837 (95% CI: 0.820–0.854), and 0.828 (95% CI: 0.810–0.846) in the validation cohort, respectively (Fig. 5A–B). Calibration plots showed excellent agreement between predicted and observed outcomes (Fig. 5C–D). Decision curve analysis confirmed clinical utility across reasonable threshold probabilities (Fig. 5E–J).

Bootstrap validation (500 resamples) yielded optimism-corrected AUC values of 0.831, 0.810, and 0.808 for 1-, 3-, and 5-year OS, closely matching the apparent performance and confirming minimal overfitting.

### 3.6. Risk stratification for clinical decision-making

**Figure 6.**
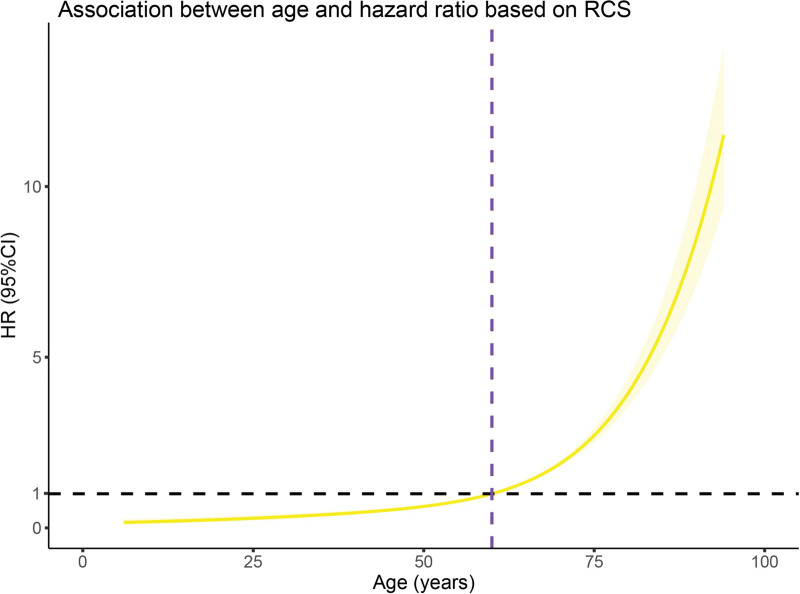
Risk stratification of CTCL patients using nomogram-derived scores. (A) Distribution of patients in the training cohort (n = 4316) shows optimal cutoff scores at 146.8 and 239.8, identified by X-tile analysis. Patients are categorized into low-risk (blue, score < 146.8), intermediate-risk (yellow, score 146.8–239.8), and high-risk (red, score > 239.8) groups. (B) The same cutoffs were applied to the validation cohort (n = 2875). Kaplan–Meier survival curves for (C) training and (D) validation cohorts confirm significantly distinct outcomes across the 3 risk groups (log-rank *P* < .0001). CTCL = cutaneous T-cell lymphoma.

Using X-tile determined cutpoints, patients were stratified into 3 distinct risk groups with significantly different survival outcomes (*P* < .0001; Fig. 6A–D). The Kaplan–Meier estimated overall survival rates at 1, 3, and 5 years in the training set were 92.5%, 86.2%, and 81.4% for the low-risk group; 72.3%, 51.1%, and 42.6% for the intermediate-risk group; and 33.8%, 21.1%, and 16.3% for the high-risk group, respectively. Consistent trends were observed in the validation set: 91.2%, 85.5%, and 80.8% (low-risk); 71.6%, 50.7%, and 41.2% (intermediate-risk); and 32.4%, 20.8%, and 15.3% (high-risk) (all log-rank *P* < .0001). These absolute risk differences underscore the clinical utility of the stratification for prognostic assessment, which may inform discussions about monitoring intensity and further evaluation.

## 4. Discussion

This study presents a prognostic nomogram for CTCL derived from population-based data. The model incorporates routinely available clinical, demographic, and treatment variables. The findings suggest that the model may have good predictive performance and appears to effectively stratified patients into groups with distinct survival outcomes, as quantified by specific survival rates at 1, 3, and 5 years.

The interpretation of our findings must account for key methodological constraints. The primary limitation is the substantial exclusion of patients due to missing data (53.7% of the initial cohort), which introduces a potential selection bias that we could not fully quantify. Direct comparison of key prognostic variables (stage, histology) between included and excluded patients was not feasible, as these fields were precisely the data missing for the excluded group. Although demographic characteristics were similar, we cannot rule out systematic differences in disease severity or other unmeasured factors. Consequently, the model may be most applicable to patients with complete records, and its broader validity requires confirmation in prospective cohorts.

A specific interpretive challenge involves the treatment variables included in the model. Their strong association with survival, particularly for chemotherapy, is best explained by confounding by indication. Consistent with this interpretation, these variables should therefore be viewed primarily as indicators of advanced disease severity rather than as independent causal factors. Throughout the manuscript, we have emphasized this distinction to prevent causal misinterpretation. The elevated HRs associated with these treatments reflect the more aggressive or advanced disease state for which these interventions are typically indicated, not an independent effect of the treatment itself.

Compared to existing tools like the Cutaneous Lymphoma International Prognostic Index index, which is restricted to specific subtypes,^[5,6]^ our model offers a structured assessment across the CTCL spectrum. However, its capacity is limited by the data available in the SEER registry, lacking detailed treatment parameters, laboratory values, and molecular biomarkers. Furthermore, the observed associations with sociodemographic factors like marital status are noted, but remain associative in nature, as direct measures of the underlying social or access-related mechanisms are absent.

The future integration of molecular data represents a critical direction for refining prognostic stratification. This is underscored by recent genomic studies that have substantially advanced our understanding of CTCL heterogeneity. Park et al identified 86 putative driver genes and demonstrated that PD-1 mutations are associated with aggressive disease behavior and worse survival.^[9]^ Similarly, integrated genomic analyses have revealed distinct molecular subtypes with differential clinical outcomes.^[10–13]^ While our SEER-based model cannot currently incorporate such parameters, future iterations that combine clinicodemographic variables with genomic features might provide more precise prognostic stratification.

From a clinical perspective, this nomogram may offer a standardized method for initial risk estimation. It may assist in identifying patients who might benefit from more intensive monitoring. Its utility lies in synthesizing multiple data points into a quantifiable risk score to inform, not replace, clinical judgment. It is important to emphasize that this nomogram is intended as a tool for risk estimation, not treatment selection. Future efforts should focus on external validation in diverse populations and on exploring the integration of molecular features.

## 5. Conclusion

A prognostic nomogram for CTCL was developed using population-based SEER data. Based on readily available clinical and demographic variables, the model demonstrated discriminative ability and generated a risk score that stratified patients into groups with significantly different survival outcomes. This standardized tool provides a practical method for initial prognostic assessment in this heterogeneous disease. Further validation in independent cohorts and future integration of molecular data are necessary steps to establish its broader clinical utility.

## Acknowledgments

Xiaojun Xu and Tiantian Sun contributed equally to the paper.

## Author contributions

**Conceptualization:** Tiantian Sun.

**Data curation:** Tiantian Sun.

**Formal analysis:** Rongli Xie, Mei Xie, Xiaojun Xu, Tiantian Sun.

**Methodology:** Rongli Xie, Mei Xie, Xiaojun Xu, Tiantian Sun.

**Resources:** Tiantian Sun.

**Writing – original draft:** Rongli Xie, Mei Xie, Tiantian Sun.

**Writing – review & editing:** Xiaojun Xu, Tiantian Sun.
